# Lap Shear and Impact Testing of Ochre and Beeswax in Experimental Middle Stone Age Compound Adhesives

**DOI:** 10.1371/journal.pone.0150436

**Published:** 2016-03-16

**Authors:** P. R. B. Kozowyk, G. H. J. Langejans, J. A. Poulis

**Affiliations:** 1 Faculty of Archaeology, Leiden University, Leiden, the Netherlands; 2 Department of Anthropology and Development Studies, University of Johannesburg, Johannesburg, South Africa; 3 Adhesion Institute, Delft University of Technology, Delft, the Netherlands; University of Oxford, UNITED KINGDOM

## Abstract

The production of compound adhesives using disparate ingredients is seen as some of the best evidence of advanced cognition outside of the use of symbolism. Previous field and laboratory testing of adhesives has shown the complexities involved in creating an effective Middle Stone Age glue using Acacia gum. However, it is currently unclear how efficient different adhesive recipes are, how much specific ingredients influence their performance, and how difficult it may have been for those ingredients to be combined to maximum effect. We conducted a series of laboratory-based lap shear and impact tests, following modern adhesion testing standards, to determine the efficacy of compound adhesives, with particular regard to the ingredient ratios. We tested rosin (colophony) and gum adhesives, containing additives of beeswax and ochre in varying ratios. During both lap shear and impact tests compound rosin adhesives performed better than single component rosin adhesives, and pure acacia gum was the strongest. The large difference in performance between each base adhesive and the significant changes in performance that occur due to relatively small changes in ingredient ratios lend further support to the notion that high levels of skill and knowledge were required to consistently produce the most effective adhesives.

## Introduction

The creation of multi-component tools was an important advancement in the history of technology, and in the evolution of the human mind [1–10]. It required the collection and combination of disparate materials in varying forms for different purposes. This is believed to have required mental capabilities analogous to those possessed by modern humans [3]. In addition, many hafted tools were held together with adhesives. Similar to the tool itself, the adhesives may have also been made using a combination of materials for different purposes [5]. Prehistoric adhesives were made out of a range of materials [11], from bitumen, a naturally occurring tar-like substance, to deciduous plant gums, conifer resins, and tars or pitches produced from the destructive distillation of birch bark and other woods [11–14]. The oldest known evidence for compound adhesives comes from the Middle Stone Age in southern Africa and may be as old as 70,000 years [15, 16]. The oldest known single-component adhesives are birch bark pitch made by Neandertals during the Middle Palaeolithic in Europe nearly 200,000 years ago [17]. The production of complex adhesives is considered to be a potential proxy for cognitive traits such as advanced working memory capacity, chronesthesia (mental time travel), multitasking, abstraction and recursion [4–8, 14, 15, 18]. A hunter’s dependency on reliable weapons would have been a strong incentive to create effective adhesives [15], and making optimised adhesive mixtures requires high levels of knowledge of natural resources to estimate ingredient ratios and understand (chemical) reactions and bonds. It also requires controlled use of fire so as not to overheat and damage the adhesive during its manufacture [5, 14, 19]. One argument for this hypothesis, that adhesive production requires modern-like cognitive abilities and a detailed understanding of the materials, is that the ratios of compound adhesive ingredients had to be very precise to successfully create glue with optimum adhesive power. This idea has not been tested systematically and the standardised adhesive property tests that we discuss in this paper are a first effort to do so.

Several previous actualistic and laboratory experiments have been conducted using replicated adhesives based on rosin (*Pinus* sp.), beeswax, ochre [20, 21], and acacia gum (*Acacia karoo* and *Acacia senegal*), [15, 19, 22]. These experiments showed that there are a number of factors that require attention for an effective adhesive to be produced and used. Allowing adhesives to air dry versus drying them near a fire, and the particle size of mineral fillers have recognisable impacts on the performance of adhesives. Our study is aimed at understanding how changing ingredient ratios influence adhesive strength. Different real-life applications of tools also subject adhesives to different load rates, and we will test several adhesive recipes with both impact and lap shear experiments to consider these changes. Laboratory testing is gaining popularity as a means to understand the materials and technologies of past human populations, and the necessity to combine actualistic field experiments with laboratory-based experiments is well understood [22–26]. In order to focus on the specific effect of changing ingredient ratios and eliminate other variables as much as possible, we opted to conduct standardised laboratory adhesive tests [27, 28], rather than field experiments.

## Materials and Methods

### Adhesive ingredients

We created 20 different adhesive recipes inspired by the archaeological record (Table 1). We experimented on commercially available pine rosin (*Pinus* sp.) and acacia gum (*Acacia senegal*) as our primary adhesives, and beeswax and red ochre powder as primary and secondary additives. All ingredients are store bought (S1 Table) to reduce as much as possible any variation that may exist in material collected from the wild. Pine rosin, otherwise known as colophony, is obtained by removing the volatile turpentine portions from pine resin [29] and was selected to represent adhesives made from conifer resins [11, 13, 30, 31]. Acacia gum was tested to compare our results with previous experiments [19, 22]. We included beeswax as the primary additive to act as a plasticiser. Beeswax may have been used approximately 40,000 years ago with resin as an adhesive [32], and shares many similarities to other lipids, such as animal or vegetable fats, possibly associated with adhesives [11, 13, 16, 33]. The use of beeswax in other experimental hafting projects also points to its possible necessity in producing successful resin-based adhesives [19–21, 34–39]. Red ochre was used as a secondary additive in combination with beeswax because of its association with adhesives and hafting among a number of different sites across Africa, Europe, and North America, and because it has been demonstrated to have positive effects on the properties of adhesives [5, 13, 19, 21, 36, 40–43]. This is a natural red iron oxide (α-Fe_2_O_3_) pigment with a particle size less than 62.5 μm from the Ardennes region, Belgium.

**Table 1:** Overview of the tested adhesive recipes.

Main Ingredient	mg	Primary Additive	mg	Secondary Additive	mg
pine rosin	250	beeswax	250	none	-
pine rosin	250	beeswax	250	ochre	50
pine rosin	250	beeswax	250	ochre	100
pine rosin	250	beeswax	250	ochre	150
pine rosin	300	beeswax	200	none	-
pine rosin	300	beeswax	200	ochre	50
pine rosin	300	beeswax	200	ochre	100
pine rosin	300	beeswax	200	ochre	150
pine rosin	350	beeswax	150	none	-
pine rosin	350	beeswax	150	ochre	50
pine rosin	350	beeswax	150	ochre	100
pine rosin	350	beeswax	150	ochre	150
pine rosin	400	beeswax	100	none	-
pine rosin	400	beeswax	100	ochre	50
pine rosin	400	beeswax	100	ochre	100
pine rosin	400	beeswax	100	ochre	150
rosin	500	none	-	none	-
acacia gum	350	beeswax	150	none	-
acacia gum	350	beeswax	150	ochre	150
acacia gum	500	none	-	none	-

### Adhesive preparation

Due to the difference in material properties, sample preparation varied somewhat between pine rosin and acacia gum adhesives. For pine rosin each ingredient was measured by weight to the nearest one-tenth of a gram and mixed together in an aluminium tray over an electric hot plate. The combined weight of rosin and beeswax in each mixture was 500 mg, and ochre was added to this in 50 mg increments (equalling 10, 20 and 30% increases). During the mixing, temperatures were kept below 140°C to avoid any thermal degradation that may take place at higher temperatures [29, 44]. Small glass beads with a diameter of 90 to 130 microns (μm) were added ‘like a pinch of salt’ and thoroughly mixed into the adhesive to ensure the set bondline thickness of each test piece was similar. These beads are often used in commercial adhesive testing in very small portions (about 2 wt%) and have no effect on the performance [45]. The adhesives were constantly stirred for two minutes before use, and again briefly in between each application on every specimen to reduce the sagging of the ingredients. Once the adhesive was completely melted and mixed, both surfaces to be bonded were simultaneously dipped in the adhesive and immediately clamped together.

Sample preparation of acacia gum was done using a method similar to Zipkin *et al*. [22]. First, the gum was ground into particles approximately 2 mm in diameter using a mortar and pestle. The appropriate amount of gum was then weighed and mixed with boiling water until it dissolved. It was then further reduced with heat until it reached a more useable consistency. The remaining ingredients were added at this point, following the same procedures used for pine rosin. Finally, unlike rosin, which behaved as a hot melt adhesive and cured as it cooled, the acacia gum required time to air dry. All samples were thus left in the open for six days (following Wadley [19]).

### Lap shear

For all material properties tests, a number of internationally recognised standards have been developed. These ensure replicability regardless of the practitioner or laboratory. One of the most common set of standards are those of ASTM International. Of these standards, lap shear tests are widely used as adhesive joint strength tests because they are easy to conduct and closely resemble the geometry of many practical joints, including one of the most common and versatile stone tool hafting methods, the cleft haft [1]. Furthermore, cutting, scraping and piercing tools must all withstand some form of shear force, in which adhesives perform best. For example, the vertical downwards force applied during cutting or scraping will create a bending stress and a vertical shear stress, and the horizontal component of the cutting force will create a tension and shear stress at the adhesively bonded joint. A piercing tool will also experience compressive shear forces on impact, and tension shear forces upon removal. As in many lap shear tests, cutting, scraping, and piercing are generally subjected to low load rates; the tool edge is placed on the worked surface, and increasing pressure is applied until there is sufficient force to cut, pierce, or scrape the surface as desired.

**Fig 1:**
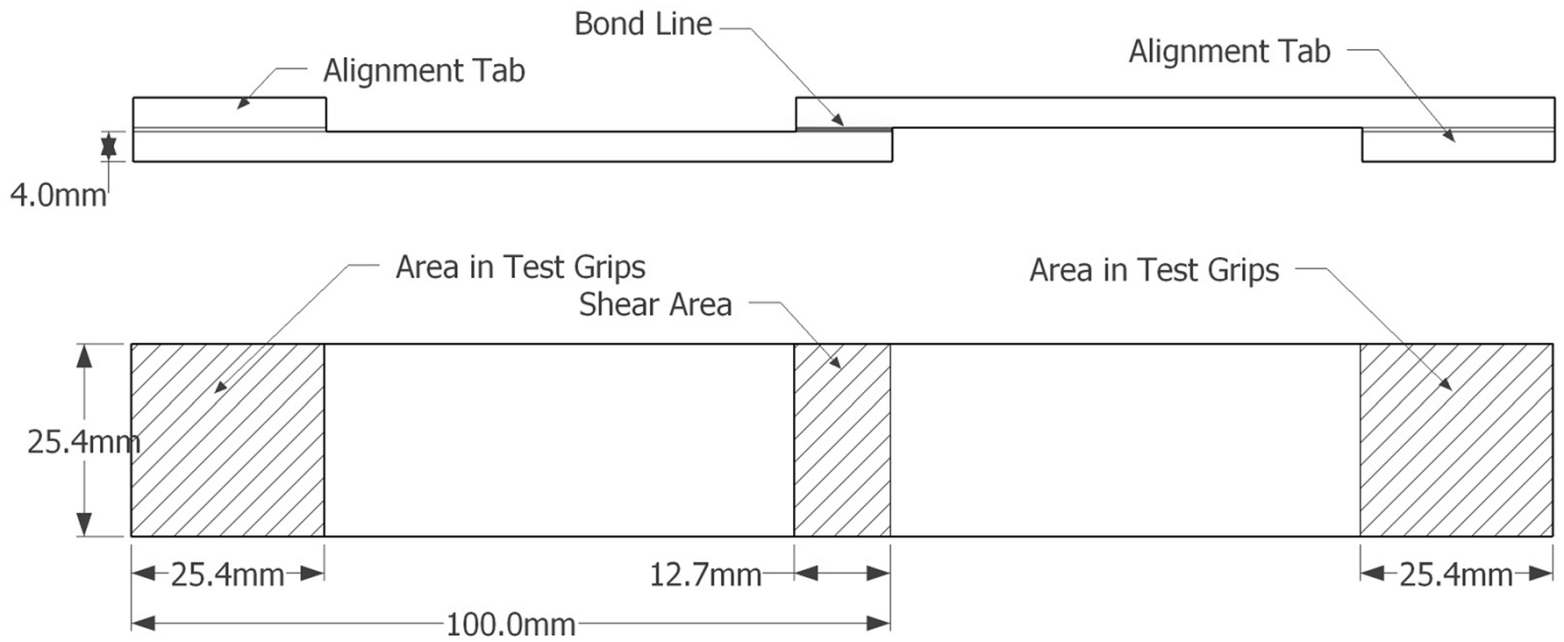
Schematic of standardised wood lap shear test specimen. Side and top view of test specimen composed of two adherends adhesively bonded together in the centre (bondline).

The ASTM D1002 test standard was therefore used for the quasi-static shear strength of a single-lap joint. This test measures ‘apparent’ shear strength because true shear strength is difficult to determine with single thin-adherend lap shear specimens, as the eccentricity of force being applied bends the substrate material and introduces peel stresses along the bond termini [46]. These additional stresses, however, help to resemble practical joints more closely, as joints in real life applications are rarely subject to perfectly planar shear forces. Due to the relatively weak nature of the adhesives (compared with modern glues) one property of the test standard was changed. We used beech (*Fagus* sp.) plywood instead of aluminium for the substrate material to improve the likelihood of cohesive failures rather than measuring bond strength of the adhesive to aluminium. The wooden test specimens are 4.0 mm × 25.4 mm × 100.0 mm long. The bond overlap was 12.7 mm (Fig 1).

**Fig 2:**
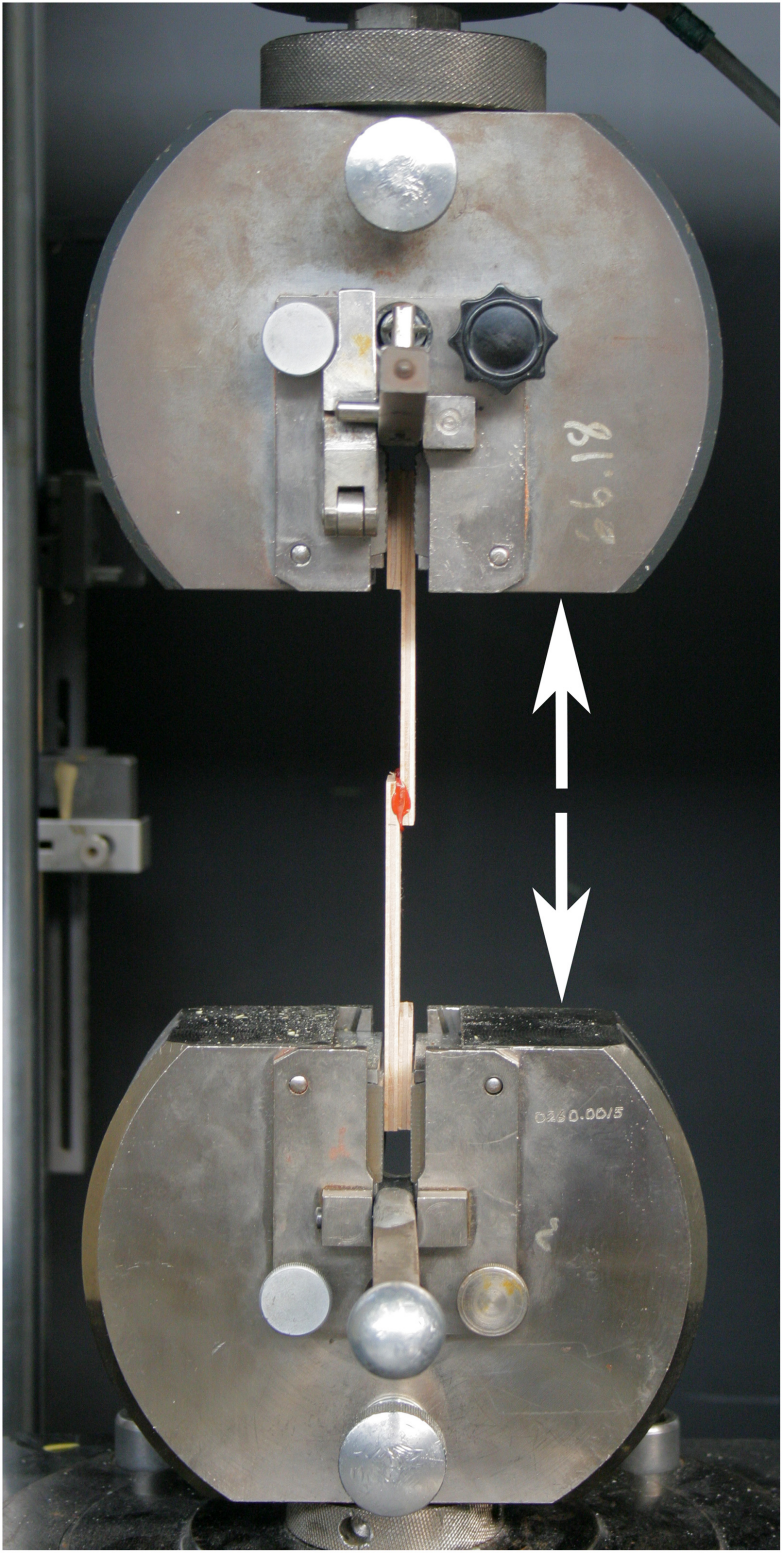
Sample in lap shear test apparatus. Clamps of a Zwick-Roell 1455 tensile bench containing standard adhesive lap shear test specimen with arrows indicating the applied direction of force.

To ensure maximum adhesion, samples were degreased with acetone, abraded with 100 grit sandpaper, and degreased again prior to the application of the adhesive. Lap shear tests were performed in the Delft Aerospace Structures and Materials Laboratory at the Delft University of Technology using a Zwick-Roell 1455 tensile bench with a 20 kiloNewton (kN) load cell at a rate of 1.3 mm/minute and a preload of 10 N. Specimens were mounted vertically between two clamps, which are then moved apart from one another at a constant speed until bond failure (Fig 2). If the adhesive does not fail completely, tests are ended automatically when the force reaches one-half that of the maximum obtained force. Five individual specimens were tested for each adhesive recipe. Tests were conducted at an ambient air temperature of 21–23°C and a relative humidity of 39–50%.

**Fig 3:**
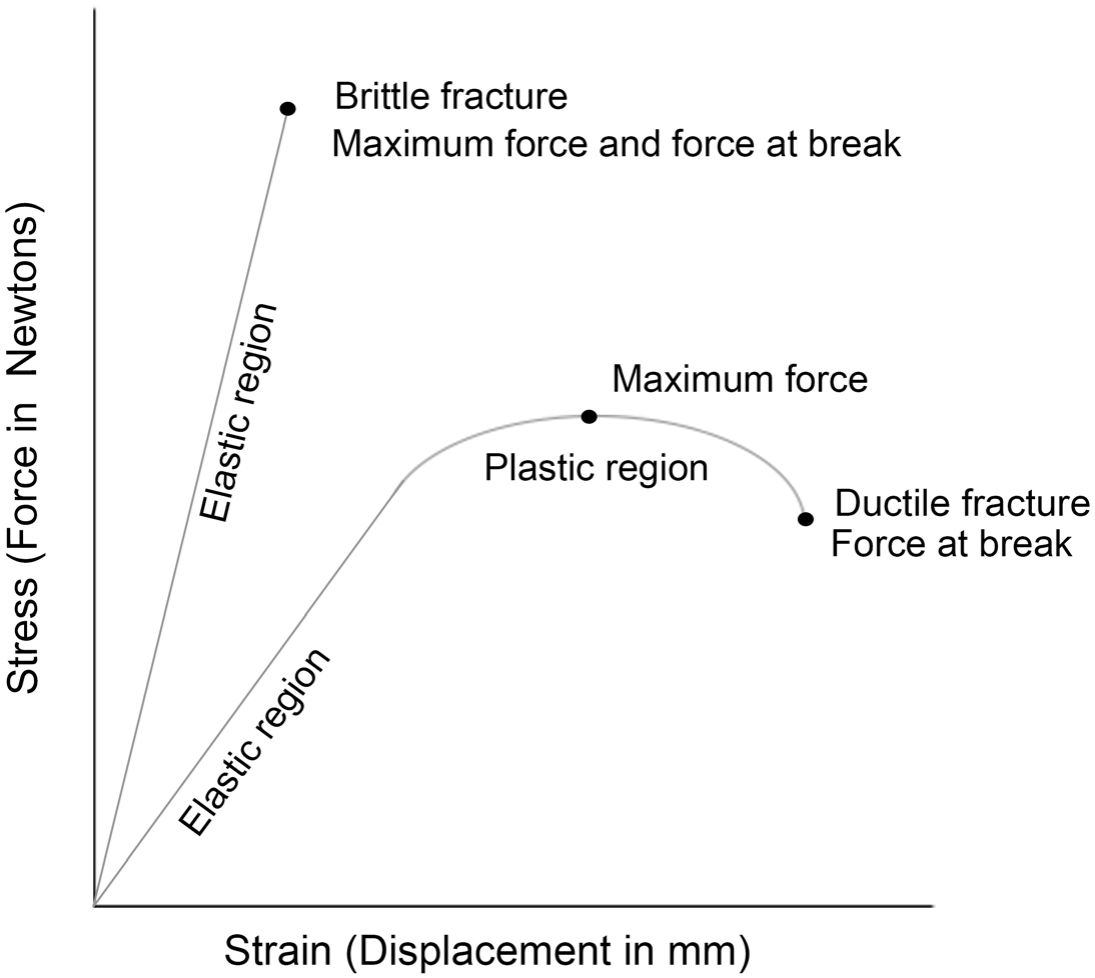
Idealised example of stress/strain curves. Curves for two different materials displaying brittle and ductile failures. The elastic region is the linear part of the curve, and any displacement along this section is temporary. The plastic region occurs after, and displacement here is permanent.

Data generated from lap shear experiments can be analyzed by two means: 1) Inspection of the bonded surfaces after failure can show if the break is adhesive or cohesive, giving essential information on the interaction between the adhesive and adherend. This is especially relevant when comparing substrate materials and adhesion strength. 2) Stress/strain curves generated by the test machine provide data on elastic and plastic deformation, brittle and ductile fracture, the maximum shear force an adhesive can withstand, and the amount a given material can be displaced before failure (Fig 3). The results are given as the maximum recorded force (N) divided by the bonded surface area (mm^2^), or Megapascals (MPa), and the displacement in mm. Fracture type can also be determined from the stress/strain curves, by looking at the amount of plastic deformation prior to absolute failure. Those curves ending abruptly with little to no arch represent brittle fractures, where the material fails catastrophically and without warning (Fig 3). Ductile fractures are shown by the gradual decrease in stress prior to failure (Fig 3). In this study, we are most concerned with the maximum force, as this is the simplest indication of what will make a strong adhesive for many different hafting purposes.

### Impact

Materials often behave differently at high load rates (impact) than they do at low load rates (quasi-static loading as in the lap shear), thus making it difficult to accurately predict how materials behave during high speed impacts based on the data obtained during low load rate tests. For example, it is possible for ductile materials to shatter abruptly under impacts [47]. High and low load rates also correspond to different prehistoric tasks; hafted spear points were probably subjected to high load rates, whereas hafted scrapers were subjected to low load rates. The load rate during ASTM D1002 lap shear test is 1.3 mm per minute (2.17 x 10^−5^ metres per second); by comparison the pendulum hammer as described in ASTM D950 impact test strikes the adherend with a velocity of 3.46 metres per second. The latter is faster than the loading speeds estimated by Shea *et al*. [48] for stabbing, but slower than those for spear throwing [48]. There are numerous procedures to test the impact resistance of materials. The most common are the Charpy and Izod tests [47], of which ASTM D950 [28] is a variant. We used this standard as guidelines to determine if some adhesive recipes are better suited to one task over another.

**Fig 4:**
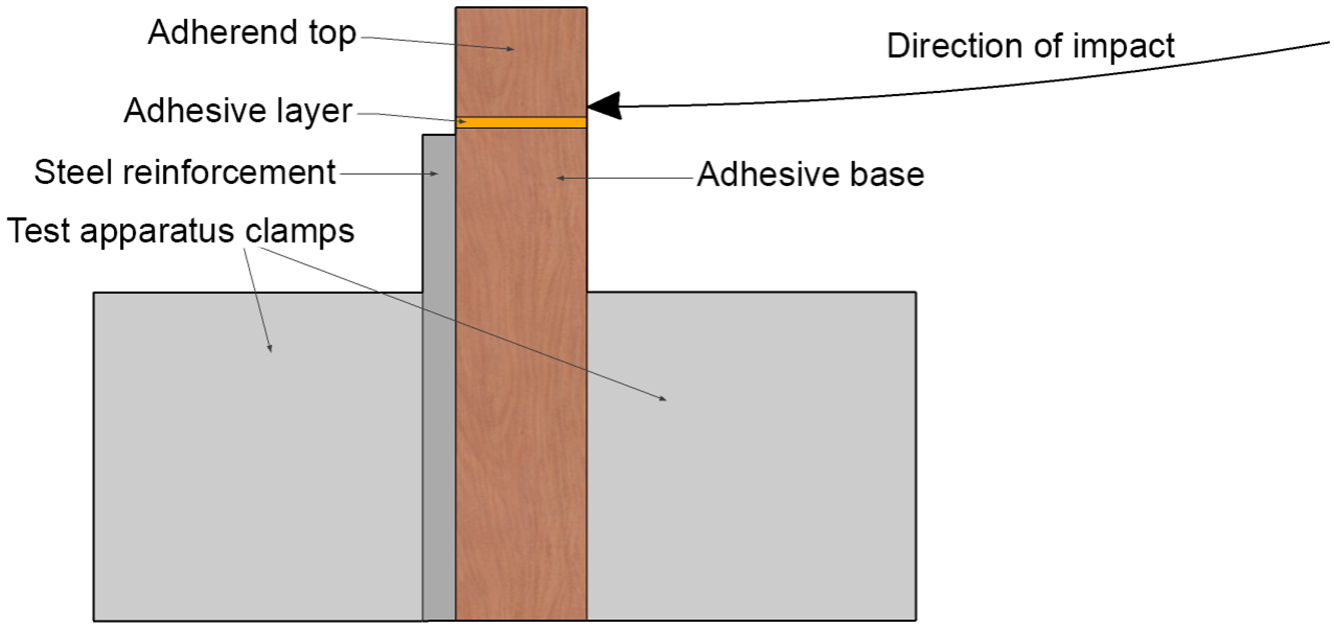
Cross section of impact test set-up. Cross section showing the direction and point of impact just above the bondline (adhesive layer) and steel reinforcement of the impact specimen.

Impact tests were performed using a Zwick 5113 pendulum impact tester in the Department of Advanced Soft Matter at the Delft University of Technology. A pendulum hammer is released from a swing angle of 124.4 degrees and accelerates to a speed of 3.46 m/s before impacting the specimen locked in the clamps. The samples were made from solid pieces of tropical hardwood, and cut to 12.0 mm × 18.0 mm × 55.0 mm. The top 10.0 mm was cut off and adhesively bonded back on with each adhesive, creating a bonded surface area of 216.0 mm^2^. The hammer impacted the 18 mm wide face of the sample less than 1 mm from the bondline. Due to test machine differences from those in the standard [28], a steel reinforcement was placed behind each specimen to ensure the adherend would not break before the adhesive (Fig 4). Impact tests were conducted at an ambient air temperature of 22–23°C and a relative humidity of 40–49%.

The absorbed impact energy in Joules (J) is recorded by measuring the difference between the maximum height of the pendulum swing before and after the impact [49]. The difference in behaviour under impact forces requires a different analysis than that of lap shear tests. The data are recorded as a single measurement of absorbed energy. The greater the energy absorbed, the better the adhesive is at withstanding impacts. Fracture type is thus not measurable, although it is assumed that most adhesives will fail in a brittle manner during impacts [47]. Adhesive and cohesive failure type can still be determined by analysing the bonded surfaces after the failure of each joint.

## Results

### Lap shear

**Fig 5:**
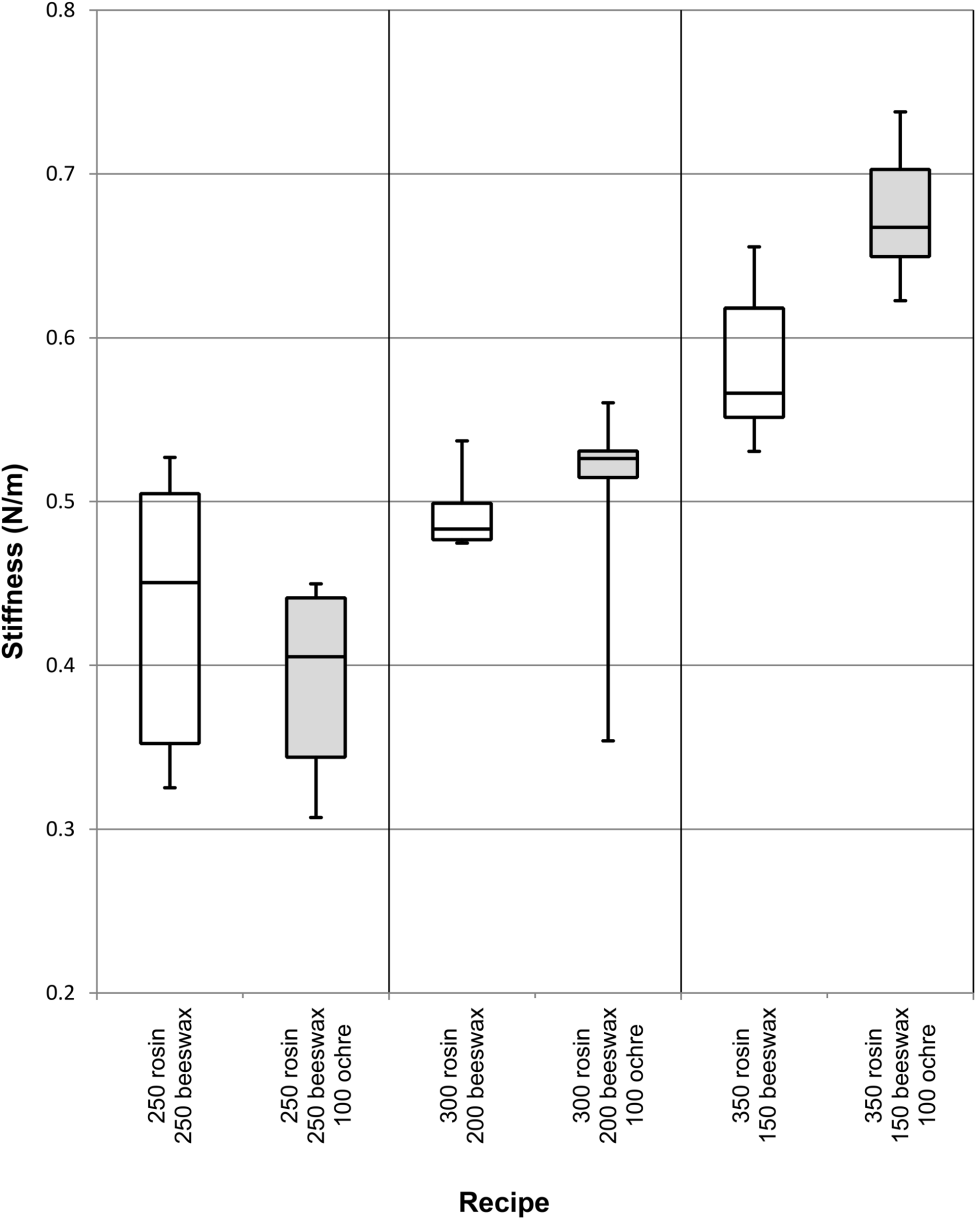
Relative stiffness of beeswax and beeswax-ochre containing adhesives. Boxplot displaying how the stiffness (N/m) of three different rosin-beeswax adhesives is affected by the addition of 100 mg ochre. Adhesive recipes are expressed by the mass of each ingredient (mg).

**Fig 6:**
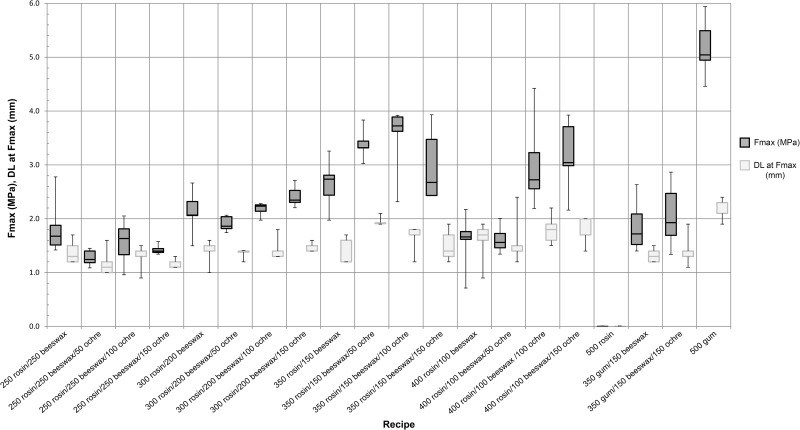
Lap shear results. Maximum force (Fmax) and displacement at maximum force (DL at Fmax) for each adhesive mixture during lap shear testing. Adhesive recipes are expressed by the mass of each ingredient (mg).

The strength of lap shear tests is recorded as the maximum force over the surface area of the bond. Table 2 displays the maximum, minimum, and mean values for each adhesive recipe. The weakest adhesive is 100% rosin; this material broke under the 10 N pre-load of the test machines and thus could not be accurately recorded. When looking at only adhesives containing rosin and beeswax, the strongest contained 350 mg rosin and 150 mg beeswax (average maximum force (Fmax) = 2.64 MPa). Adding ochre in 50 mg increments to this adhesive further improved the performance. The strongest adhesive using rosin contains 350 mg rosin, 150 mg beeswax and 100 mg red ochre powder (average Fmax = 3.49 MPa). Moreover, when no ochre is present, the 350 mg rosin/150 mg beeswax adhesive becomes significantly weaker than that containing the optimum amount of ochre (P = 0.05, two-tailed t-test). The mean of the next five strongest rosin-beeswax-ochre adhesives all fall within the range of the 350 mg rosin/150 mg beeswax/100 mg ochre mixture. By dividing the maximum force (N) by the total displacement (mm) of two adhesives, an approximation of stiffness (N/m) can then be compared. In the correct proportions (350 mg rosin/150 mg beeswax/100 mg ochre), ochre improves the stiffness of adhesive mixtures. However, with higher beeswax-containing adhesives (200 mg and 250 mg beeswax), adding 100 mg ochre has no measurable effect on stiffness (Fig 5). The weakest rosin adhesive contains 250 mg rosin, 250 mg beeswax and 50 mg ochre (average Fmax = 1.297 MPa). The strongest adhesive overall is made of 100% acacia gum (average Fmax = 5.18 MPa). Beeswax only, and beeswax and ochre combinations reduce the average strength of pure acacia gum to 1.87 MPa and 2.06 MPa, respectively. Adhesive maximum force and displacement at maximum force for each recipe is presented in Fig 6.

**Table 2:** Overview of lap shear results.

Recipe (mg)	Mean Fmax Mpa	S	Maximum Fmax	Minimum Fmax	Mean DL at Fmax	S
250 rosin/250 beeswax	1.85	0.55	2.78	1.42	1.4	0.2
250 rosin/250 beeswax/50 ochre	1.27	0.15	1.45	1.09	1.2	0.3
250 rosin/250 beeswax/100 ochre	1.56	0.43	1.81	0.96	1.3	0.2
250 rosin/250 beeswax/150 ochre	1.43	0.09	1.58	1.34	1.1	0.1
300 rosin/200 beeswax	2.12	0.43	2.66	1.50	1.4	0.2
300 rosin/200 beeswax/50 ochre	1.91	0.14	2.07	1.74	1.4	0.1
300 rosin/200 beeswax/100 ochre	2.18	0.13	2.28	1.97	1.5	0.2
300 rosin/200 beeswax/150 ochre	2.42	0.20	2.71	2.20	1.5	0.1
350 rosin/150 beeswax	2.64	0.47	3.26	1.97	1.5	0.3
350 rosin/150 beeswax/50 ochre	3.39	0.29	3.44	3.02	1.9	0.1
350 rosin/150 beeswax/100 ochre	3.49	0.67	3.92	2.32	1.6	0.3
350 rosin/150 beeswax/150ochre	2.99	0.68	3.93	2.43	1.5	0.3
400 rosin/100 beeswax	1.59	0.53	2.17	0.71	1.6	0.4
400 rosin/100 beeswax/50 ochre	1.62	0.26	2.01	1.34	1.6	0.5
400 rosin/100 beeswax/100 ochre	3.02	0.87	4.42	2.19	1.8	0.3
400 rosin/100 beeswax/150 ochre	3.17	0.69	3.92	2.16	1.8	0.2
500 rosin	-	-	-	-	-	-
350 acacia gum/150 beeswax	1.87	0.50	2.63	1.40	1.3	0.1
350 acacia gum/150 beeswax/150 ochre	2.06	0.61	2.87	1.34	1.4	0.3
500 acacia gum	5.18	0.56	5.94	4.46	2.2	0.2

Mean maximum force (Fmax), maximum Fmax, minimum Fmax, displacement (DL) at Fmax, and standard deviations (S) of all lap shear tests (n = 5 for each recipe). Adhesive recipes are expressed by the mass of each ingredient (mg).

**Fig 7:**
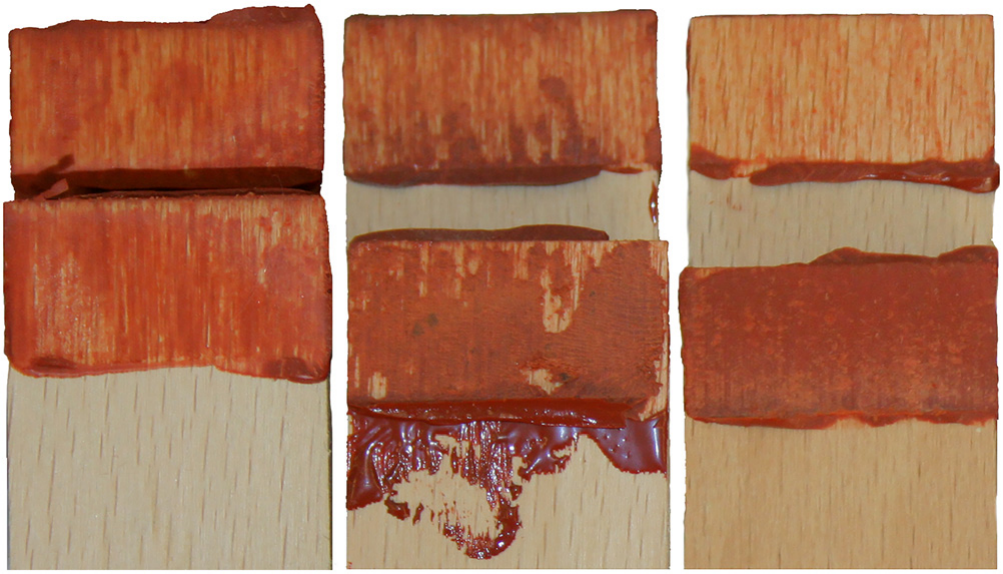
Example of failure modes through examination of the bonded surfaces after lap shear test completion. Left: cohesive (250 mg rosin/250 mg beeswax/50 mg ochre), the adhesive remains evenly bonded to both sides; middle: mixed-mode (400 mg rosin/100 mg beeswax/100 mg ochre), the adhesive favours one side, but remains bonded to both some areas; right: adhesive failure (400 mg rosin/100 mg beeswax/50 mg ochre), the adhesive remains bonded to one side only.

Understanding the failure mode is important for adhesive tests, as it helps indicate which property is being measured. A cohesive failure measures the intermolecular bond strength within the adhesive, while adhesive failures measure the bond between the adhesive and the adherend. With the exception of the 250 mg rosin/250 mg beeswax recipe, which failed adhesively, most other failures were either mixed mode or cohesive (Table 3). However, the classification of failure type on wood lap shear tests proved to be difficult because of the porosity of the wood. Even failures that appeared primarily adhesive still exhibited some evidence of cohesive failure because of the separation of adhesive material with that still present inside the pores of the wooden surface. This was further complicated when ochre was added, as the staining of the wood made it more difficult to separate adhesive failure from cohesive failure. These problems reduced the number of fully diagnostic adhesive failures. Mixed mode failures typically exhibit signs of both cohesive and adhesive failures, and are therefore highly prevalent due to the aforementioned difficulties (Fig 7). There is also a shift among fracture types in rosin adhesives where those ≥350 mg rosin exhibit more brittle fractures and those under <350 mg rosin fail in a ductile manner (Table 3).

**Table 3:** Overview of failure modes and fracture types from all lap shear tests.

Recipe (mg)	Cohesive Failure	Adhesive Failure	Mixed Mode Failure	Brittle Fracture	Ductile Fracture
250 rosin/250 beeswax		5		5	
250 rosin/250 beeswax/50 ochre	3		2		5
250 rosin/250 beeswax/100 ochre	2		3		5
250 rosin/250 beeswax/150 ochre	1		4		5
300 rosin/200 beeswax	5			4	1
300 rosin/200 beeswax/50 ochre	1		4		5
300 rosin/200 beeswax/100 ochre	1		4		5
300 rosin/200 beeswax/150 ochre	3		2		5
350 rosin/150 beeswax	2		3	5	
350 rosin/150 beeswax/50 ochre	2		3	4	1
350 rosin/150 beeswax/100 ochre	3		2	5	
350 rosin/150 beeswax/150ochre		1	4	5	
400 rosin/100 beeswax			5	5	
400 rosin/100 beeswax/50 ochre		3	2	5	
400 rosin/100 beeswax/100 ochre	3		2	5	
400 rosin/100 beeswax/150 ochre			5	5	
500 rosin	-	-	-	-	-
350 acacia gum/150 beeswax	5			5	
350 acacia gum/150 beeswax/150 ochre			5	5	
500 acacia gum	5			5	
**Total**	**36**	**9**	**50**	**63**	**32**

Most failures are either cohesive or mixed-mode, suggesting the property being measured was the cohesive strength of the adhesive, and not purely the bond strength to the substrate. Adhesives with <350 mg rosin tend to fail in a ductile manner, while those with ≥350 mg rosin tend to fail in a brittle manner. n = 5 for each recipe. Adhesive recipes are expressed by the mass of each ingredient (mg).

### Impact

**Fig 8:**
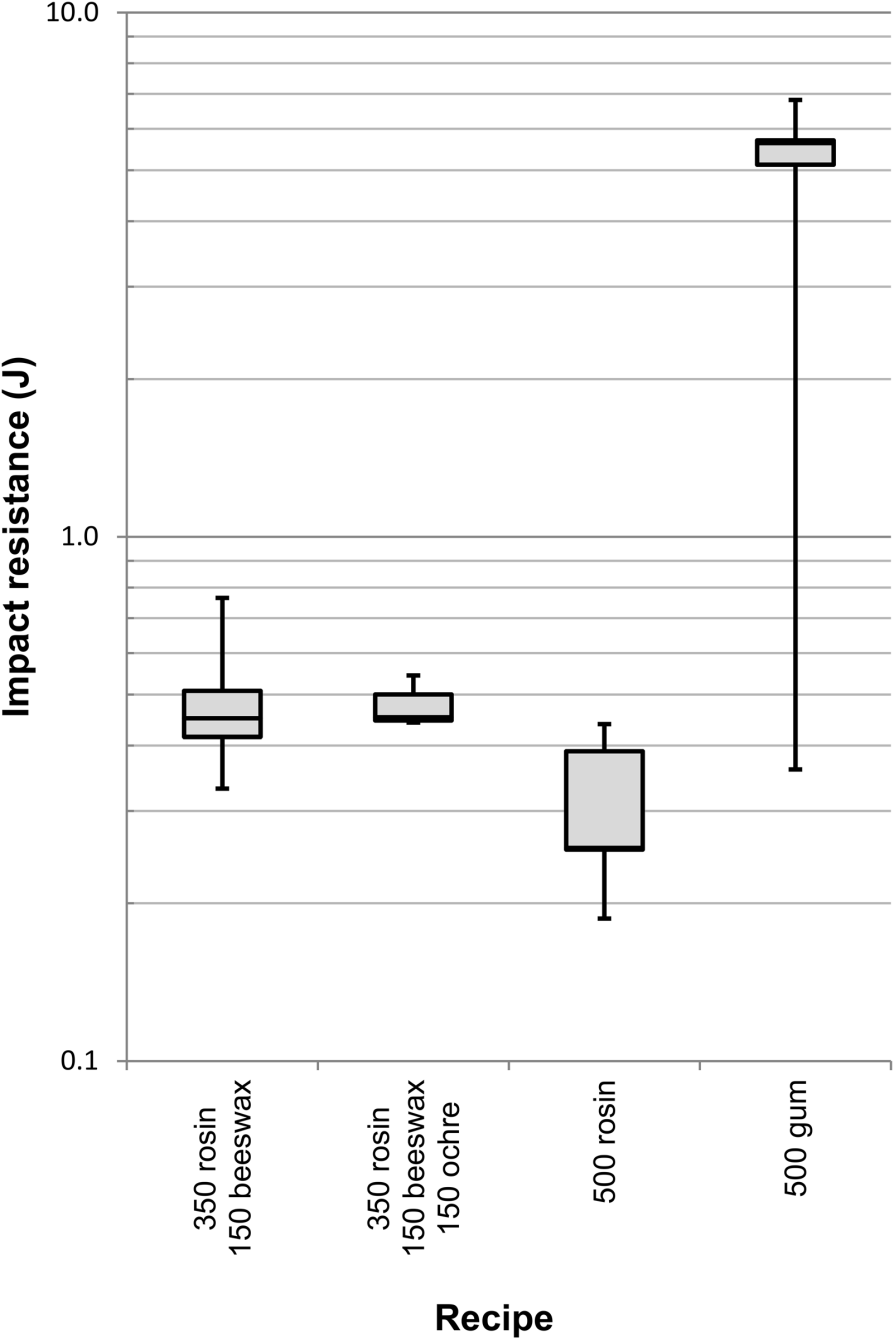
Impact test results. The logarithmic y-axis represents impact resistance in Joules for each recipe. Adhesive recipes are expressed by the mass of each ingredient (mg).

**Fig 9:**
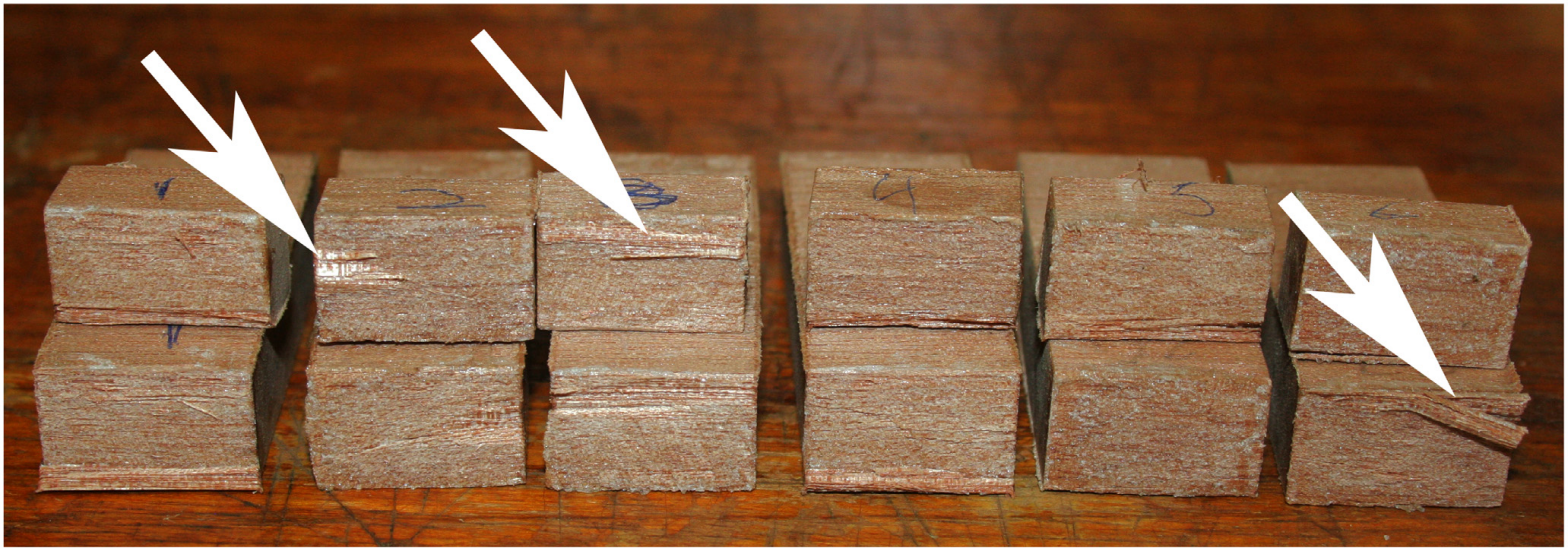
Photograph showing bonded surfaces of wood adherends with 100% acacia gum after the impact tests. All specimens exhibit some form of substrate failure, though some are more severe than others. The arrows point to areas where the wood failed but the adhesive remained bonded.

The aim of the impact tests was to determine how much each base adhesive was affected by high load rates. Impact resistance is a measure of the adhesive’s ability to withstand a rapid application of force. This represents a different practical use of composite tools compared to lap shear tests. Table 4 displays the mean, maximum, minimum and standard deviation of each recipe tested for impact resistance, and Fig 8 shows them in relation to one another. The adhesive consisting of pure rosin was weaker than adhesive mixtures with beeswax and beeswax-ochre (average impact resistance of 0.31 J versus 0.48 J and 0.48 J respectively). One hundred percent acacia gum remained the strongest adhesive and had an average impact resistance of 5.75 J, more than ten times stronger than any rosin adhesive. In addition, the recorded impact resistance for acacia gum was limited in part by the strength of the substrate material and not the adhesive, because in every instance (n = 6) the wood specimens broke on or very near the bondline (Fig 9).

**Table 4:** Overview of impact test results.

Recipe (mg)	Mean Impact Resistance	Max Impact Resistance	Min Impact Resistance	S	n
350 rosin/150 beeswax	0.48	0.76	0.33	0.13	8
350 rosin/150 beeswax/150 ochre	0.48	0.54	0.44	0.04	6
500 rosin	0.31	0.44	0.19	0.11	5
500 acacia gum	4.85	6.82	0.36	0.67	6

Mean, maximum, minimum, and standard deviation (S) of impact resistance (J) for each recipe. Adhesive recipes are expressed by the mass of each ingredient (mg).

During practical use in prehistoric hafting, the adhesive may have acted as more of a plastic to fill spaces and irregularities between the stone insert and the handle, keeping it in place mechanically rather than adhesively. In such cases, measuring the cohesive strength becomes more important. None of the impact tests resulted in adhesive failures. The 350 mg rosin/150 mg beeswax/150 mg ochre adhesive contained one instance of a mixed mode failure, and all the others were cohesive failures, suggesting that the weakest point during impact is the adhesive material itself, and not the bond strength between the two materials.

## Discussion

Lap shear experiments with rosin and beeswax performed as expected and support the findings reported in previous studies [19, 21, 29]. Beeswax greatly improves the performance by reducing brittleness, and changes of as little as 50 mg (10%) can reveal measurable differences in maximum force and stiffness. However, during the lap shear experiments the optimum ingredient ratio was considerably different to that identified by Gaillard *et al*. [20] under projectile impact experiments. Their results indicate that a ratio of 30% rosin to 70% beeswax is optimum. This difference may be a result from different joint geometries being tested. In our single lap shear joint tests, recipes containing 50% beeswax failed adhesively. If the adhesive was filling an uneven space (e.g. those of Gaillard *et al*., *p*.*5* [20]), which would result in more of a mechanical bond holding the flint in place, rather than being between two flat and parallel surfaces, the performance of the higher beeswax content adhesives may improve. This difference compliments the idea that specific adhesive recipes may be required for different tasks, or different haft types, as one type of joint and application of force produces different final results.

**Fig 10:**
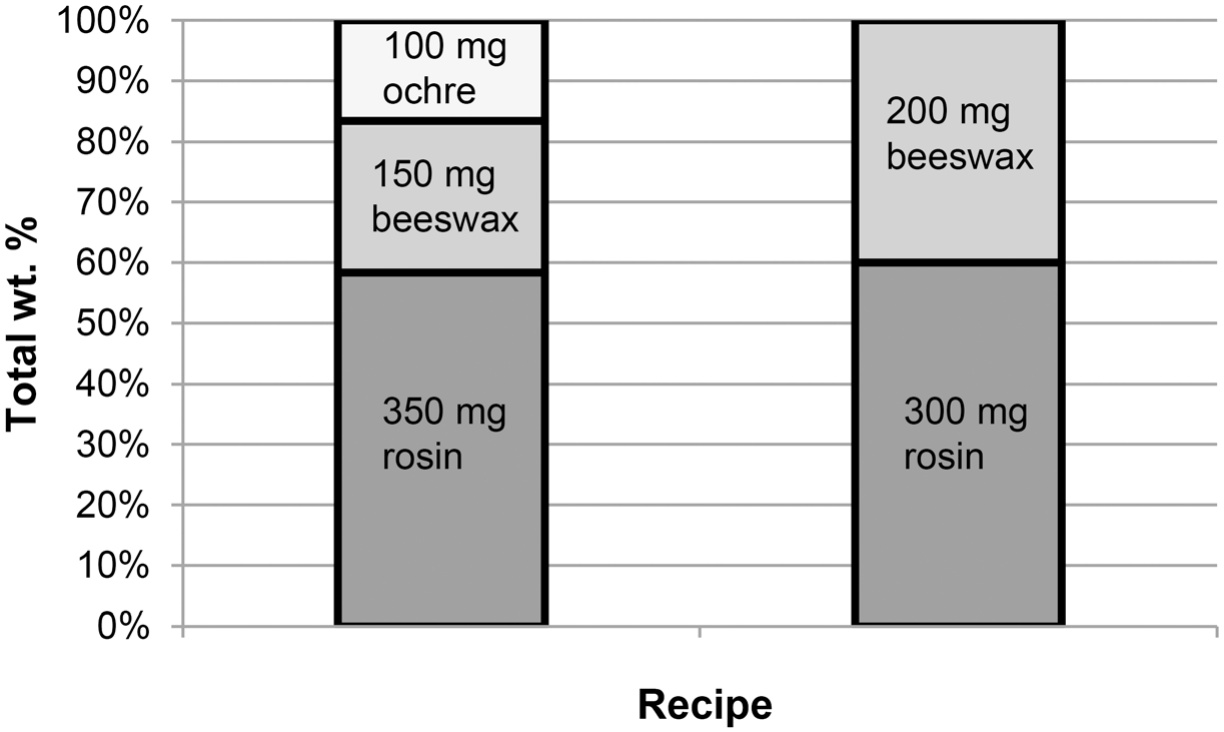
Comparison of wt% ratios for two different recipes.

The addition of ochre as a third ingredient does not have a one-to-one relationship with performance and does not simply improve each mixture to a certain degree depending on its amount. For example, although it improved the performance in rosin-beeswax mixtures containing ≤30% (150 mg) beeswax, when ochre was added to recipes containing >30% beeswax the resulting adhesive withstood less static force than when no ochre was present. Theoretically, this can be explained by the ratio of rosin to additives. Rosin provides much of the ‘tack’, holding everything together and sticking to the substrate surfaces, but requires beeswax to prevent it from cracking, and ochre to further stiffen it. Mixtures containing 60% (300 mg) or less rosin are already short on ‘tack’, and the addition of ochre further reduces the overall amount of rosin, weakening the adhesive even more. However, the ratio of rosin to total weight percentage (wt%) is not the only thing affecting the strength of the adhesive. Ochre was added as an addition to an already blended rosin-beeswax mixture, so 350 mg rosin/150 mg beeswax/100 mg ochre actually contained a smaller rosin-to-total ratio than 300 mg rosin/200 mg beeswax with no ochre, but performed significantly better (P < 0.01, two tailed t-test; Fig 10). To summarise, the first step in the process must be correct for the second ingredient to work effectively; add too much beeswax to begin with, and ochre will harm the performance of the adhesive. This suggests that not only is precision required to create the optimum rosin-to-beeswax ratio, but the addition of ochre may require more forward planning if it were to be used efficiently.

As adhesives often play an important part as fillers in a haft, high plastic deformation is a negative trait. We initially hypothesised that adhesives that showed plastic deformation and subsequent warning prior to ductile fractures were beneficial, as preventative measures can often be taken to stop complete failure [47]. Ductile fractures also do not result in the same amount of material loss as brittle fractures do, as the latter typically shatters into smaller fragments. In a situation where resources might have been scarce or time-consuming to prepare, preventing absolute failure may have been more important than the maximum strength. However, if an adhesive can withstand a maximum force greater than that which was ever applied to it during use, without undergoing any plastic deformation, then it would remain in its original position after each time it was used. The stone insert would thus be prevented from becoming loose and breaking away from the haft for an extended period of time. Once a material undergoes plastic deformation, however, its shape will be permanently altered. In the context of a hafted stone tool, this may be just enough to create an uneven coverage of either the stone implement, or the wooden handle, creating wiggle room, pressure points, or leverage; all of these can expedite the failure of the haft, and might even necessitate the breaking of the handle or stone insert. Furthermore, tools such as spears would not be very efficient if the point was easily pushed permanently out of alignment. It would therefore be beneficial to determine, through more experimentation, what maximum forces are applied during practical hand-held uses of different tools.

Experimentation by impact testing of pure rosin, rosin-beeswax, and rosin-beeswax-ochre adhesives was conducted to provide a brief comparison of how these recipes perform under different load rates [50]. In general, the performances of the impact tests support those of the lap shear tests. That is, compound rosin adhesives perform better than single component rosin adhesives, and pure acacia gum is the strongest. However, the difference between rosin based adhesives was much less pronounced under impact than lap shear forces. Pure rosin was too brittle and weak to be used in our lap sheer tests, as it broke under the preload of the test machine. However, the performance during impact resistance tests, coupled with examples of resins being used pure from ethnohistoric and archaeological sources [13, 51, 52] suggests that, although not ideal, pure rosin may still be used successfully for certain applications. For example, if the purpose was to create an adhesive that would shatter on impact thus dislodging the flint point potentially causing more soft tissue damage to the target [19, 51], pure rosin may be preferable.

Acacia gum does not need any additives and performs exceptionally well under both load types. This is interesting, given previous results from actualistic experiments [6, 19] in which pure acacia gum was said to be more brittle and weaker than mixtures containing ochre and beeswax. This may result from a different type or origin of the gums used, different environmental conditions, or it may be a result of joint geometry more than adhesive properties. Wadley has shown that pure natural gum adhesives are weak under damp or wet conditions [19]. In these situations, additives such as ochre, beeswax or fat may have a different effect on performance. The joints Wadley [6, 19] used were large balls of adhesive that acted more like a plastic surrounding the stone insert. Our lap shear and impact tests contain only a thin adhesive layer between two flat and well-fitting substrates. Wadley [19] recorded the pure acacia gum adhesives as containing lots of air bubbles and cracks, which crumbled during use. This is less of a problem when the adhesive is applied in a thin layer and clamped. Not only will air bubbles be forced out during clamping and escape a thin layer more easily, the thin layer also reduces the volume of adhesives that may contain large air pockets or defects, thus theoretically reducing the likelihood of weak spots where crack propagation may take place.

The skill required to produce the best adhesive itself is not the only difficult part of creating an efficient haft. Particular adhesives may be better suited to particular joint geometries. The surface preparation and joint assembly must also be accounted for. Surface preparation greatly influences the performance of an adhesive joint [22, 46]. Any defects along the bondline, particularly near the bond termini, can severely weaken the performance. It follows that if a haft were to be poorly constructed and contained sharp notches, defects, and large spaces ‘filled’ with adhesive, the strength could be significantly compromised. Although it appears common sense to create smooth edges around a stone tool insert, and we may presently be predisposed to do so for aesthetic reasons, this adds a level of ‘folk engineering’ to the construction of hafts. Barham [1] has already suggested it is likely that the early inventors of hafted tools understood the ‘folk physics’ of different forces on different tools, such as compression, tension and shear. They would have understood that the haft is the weakest part of the tool, and found ways to improve its strength [1]. One of these ways was to reduce any point where stresses could concentrate and crack propagation can start. As a consequence, the ‘workability’ of the material becomes more important in manufacturing a strong haft. A material that is hard to work with, even if stronger than another, may ultimately result in a weaker joint because it contains more defects due to poor application. Lithic standardization and the production of less irregularly shaped artefacts may be another approach to solving this problem. Adhesive performance could be ‘improved’ by creating a tool that is easier to haft and glue in a clean and smooth manner.

The situations in which acacia gum adhesives broke the wood substrate material during impact tests raise another possibility relating to the addition of ochre and beeswax to some adhesive mixtures. Wooden handles require a considerable investment in time and effort, and it has been suggested that they were re-used [53]. Stone tools could also be removed from a haft, re-sharpened, and then re-attached [1, 53, 54]. An adhesive that outlasts both the stone tool and the wooden handle might not be as efficient as one which fails before the other components of the tool. It may be more of an investment to replace a wooden handle than a small amount of adhesive. It is possible that ochre and/or beeswax were added to create a softer and weaker adhesive mixture that would reduce the damage caused to a handle or insert. Furthermore, unlike rosin, which melts easily at low temperatures, dry acacia gum requires crushing and dissolving in hot water before it can be re-used. The addition of beeswax or fat may allow the adhesive to be softened at a lower temperature, facilitating an easy removal of a dull or broken stone insert. More research is required on the effect of additives to specific physical properties of adhesives, such as melting point and tool re-use to validate such hypotheses.

Although the rosin results described above, that ochre as filler and beeswax as a plasticiser can be used to improve the performance of an adhesive, are in agreement with other studies [6, 21], there is one main difference that should be pointed out relating to *how* ochre improves the performance. Allain and Rigaud [21] reported that ochre helps blend resin (rosin) and beeswax, creating a more homogenous mixture. However, it has since been shown that one of the benefits of adhesives made from rosin and beeswax is the natural miscibility of the two ingredients [50]. As a result, they work very well together, specifically because of their ability to blend easily and completely with one another. Acacia gum, although water soluble, has what is known as an ‘arabinogalactan protein fraction’, which orients oils and makes it naturally able to blend water and lipids. For this reason, acacia gum is employed as an emulsifier to blend ingredients of food-stuffs today, such as water-based drinks with oil-based flavour components [55–57]. It is therefore unlikely that ochre was included to help blend resin or acacia gum with lipid plasticising agents. However, it is still possible that other plant gums potentially collected by MSA humans may not have had this property, and consequently required an ochre-like emulsifying agent.

In a natural setting, the properties of the adhesive ingredients will not be as consistent as our contemporary store-bought counterparts, and the real life applications can vary beyond lap shear and impact test. This is where the ‘artisanship’ of the tool maker comes in [6]. Our results indicate how some specific recipes out-perform others, but to achieve similar results with natural products, many other factors need to be taken into account, understood, and adjusted for. Ochre can vary in quality from one location to another, gum and resin can be affected by exposure time to the air and sun, seasonality and even the previous year’s climate [6, 58–60]. As shown in our experiments, adhesive efficacy is sensitive to small recipe changes, and this affirms the idea that adhesive manufacturers were ancient artisans [6]. Moreover, it supports the hypothesis that they had the procedural knowledge and cognitive prerequisites necessary for the complex production of compound adhesives, including an understanding of plasticity, consistency, adhesion, and the ability to use abstract reasoning and forward planning. (cf. [6, 8]).

## Conclusion

Lap shear and impact experiments using different base adhesives and different combinations of additives have successfully shown that changes by as little as 10 wt%. beeswax and ochre can measurably improve performance, but too much will decrease the strength of the adhesive. The addition of beeswax in the correct proportions reduces brittleness, resulting in a stronger adhesive, and ochre can further strengthen the adhesive and will create a stronger and stiffer material, but only in the correct combination with beeswax. Ochre and beeswax improve the impact resistance of pure rosin, but to a lesser degree than they improve lap shear strength. Under the circumstances tested here, pure acacia gum is the strongest adhesive, and unlike rosin it is weakened by the addition of beeswax and ochre. However, the optimum ratio of ingredients is not universal for different base adhesives, or for different tool types and applied forces.

The significant changes that occur in adhesive properties due to small changes in material ratios or manipulations, as demonstrated by the addition of beeswax and ochre to rosin and gum, clearly indicates how intricate adhesive technology is. Rosin-based compound adhesives are challenging to get 'just right', and require precise changes to the ingredients. Considerable technical skill with fire would also be required to melt or dry rosins and gums without burning them [6]. Further on-the-spot adjustments to ingredients and ingredient ratios would also be required to compensate for how differently rosin and gum adhesives react to additives. Mental rotation, abstract thinking, forward planning and a detailed understanding of natural adhesive material properties and how they combine would therefore have been required by MSA people to create effective compound adhesives [6, 15, 19].

Our results have further demonstrated the wide range of performance properties available from prehistoric adhesives, and their possible suitability for different uses. When the combinative effects of ingredients and additives are considered along with the number of different materials associated with adhesive use and hafting [3, 5, 11, 13, 30], the implied capacity for creative thinking, knowledge, and skill is further increased (confirming [6]). However, as direct evidence of adhesives from the Middle Palaeolithic and Middle Stone Age is still relatively sparse, additional research will greatly improve our understanding of these materials. Such studies include analysing the preservation qualities, chemical identification and quantification of adhesive components, and more standardized performance testing of different adhesives and joints. All of these research areas will provide additional insight into the purpose of specific materials and material combinations, and will thus contribute to a better understanding of the early humans who used them.

## Supporting Information

S1 Table Ingredient information. All ingredients were purchased from Kalverringdijk 29, 1509BT Zaandam, NL. Tel: +31(0)75 621 0477. Website: http://www.verfmolendekat.com/webshop/
(DOCX)

## References

[1] Barham L. From Hand to Handle: The First Industrial Revolution. Oxford: Oxford University Press; 2013.

[2] Ambrose SH. Paleolithic technology and human evolution. Science. 2001;291(5509):1748–53. 10.1126/science.1059487 11249821

[3] Ambrose SH. Coevolution of composite‐tool technology, constructive memory, and language. Current Anthropology. 2010;51(s1):S135–S47. 10.1086/650296

[4] Haidle MN, Bolus M, Collard M, Conard NJ, Garofoli D, Lombard M, et al The nature of culture: an eight-grade model for the evolution and expansion of cultural capacities in hominins and other animals. Journal of Anthropological Sciences. 2015. 10.4436/JASS.93011 26196109

[5] Lombard M. The gripping nature of ochre: The association of ochre with Howiesons Poort adhesives and Later Stone Age mastics from South Africa. Journal of Human Evolution. 2007;53(4):406–19. 10.1016/j.jhevol.2007.05.004 . 17643475

[6] Wadley L. Compound‐adhesive manufacture as a behavioral proxy for complex cognition in the Middle Stone Age. Current Anthropology. 2010;51(s1):S111–S9. 10.1086/649836

[7] Wadley L. Recognizing complex cognition through innovative technology in Stone Age and Palaeolithic sites. Cambridge Archaeological Journal. 2013;23(02):163–83. 10.1017/s0959774313000309

[8] Wynn T. Hafted spears and the archaeology of mind. Proceedings of the National Academy of Sciences of the United States of America. 2009;106(24):9544–5. 10.1073/pnas.0904369106 19506246 PMC2701010

[9] Wynn T. Archaeology and cognitive evolution. The Behavioral and brain sciences. 2002;25(03):389–402. 12879699 10.1017/s0140525x02000079

[10] McBrearty S, Brooks AS. The revolution that wasn't: A new interpretation of the origin of modern human behavior. Journal of Human Evolution. 2000;39(5):453–563. 10.1006/jhev.2000.0435 . 11102266

[11] Regert M. Investigating the history of prehistoric glues by gas chromatography-mass spectrometry. Journal of separation science. 2004;27(3):244–54. 10.1002/jssc.200301608 . 15334911

[12] Boëda E, Bonilauri S, Connan J, Jarvie D, Mercier N, Tobey M, et al Middle Palaeolithic bitumen use at Umm el Tlel around 70 000 BP. Antiquity. 2008;82(318):853–61.

[13] Helwig K, Monahan V, Poulin J, Andrews TD. Ancient projectile weapons from ice patches in northwestern Canada: Identification of resin and compound resin-ochre hafting adhesives. Journal of Archaeological Science. 2014;41:655–65. 10.1016/j.jas.2013.09.010

[14] Koller J, Baumer U, Mania D. High-Tech in the Middle Palaeolithic: Neandertal-manufactured pitch identified. European Journal of Archaeology. 2001;4(3):385–97. 10.1177/146195710100400315

[15] Wadley L, Hodgskiss T, Grant M. Implications for complex cognition from the hafting of tools with compound adhesives in the Middle Stone Age, South Africa. Proceedings of the National Academy of Sciences of the United States of America. 2009;106(24):9590–4. 10.1073/pnas.0900957106 19433786 PMC2700998

[16] Lombard M. First impressions of the functions and hafting technology of Still Bay pointed artefacts from Sibudu Cave. Southern African Humanities. 2006;18(1):27–41.

[17] Mazza PPA, Martini F, Sala B, Magi M, Colombini MP, Giachi G, et al A new Palaeolithic discovery: Tar-hafted stone tools in a European Mid-Pleistocene bone-bearing bed. Journal of Archaeological Science. 2006;33(9):1310–8. 10.1016/j.jas.2006.01.006

[18] Wragg Sykes RM. To see a world in a hafted tool: birch pitch composite technology, cognition and memory in Neanderthals In: Coward F, Hosfield R, Pope M, Wenban-Smith F, editors. Settlement, Society and Cognition in Human Evolution: Cambridge University Press; 2015.

[19] Wadley L. Putting ochre to the test: Replication studies of adhesives that may have been used for hafting tools in the Middle Stone Age. Journal of Human Evolution. 2005;49(5):587–601. 10.1016/j.jhevol.2005.06.007 . 16126249

[20] Gaillard Y, Chesnaux L, Girard M, Burr A, Darque-Ceretti E, Felder E, et al Assessing Hafting Adhesive Efficiency in the Experimental Shooting of Projectile Points: A new Device for Instrumented and Ballistic Experiments. Archaeometry. 2015:n/a–n/a. 10.1111/arcm.12175

[21] Allain J, Rigaud A. Décor et fonction. Quelques exemples tirés du Magdalénien. L'Anthropologie. 1986;90(4):713–38.

[22] Zipkin AM, Wagner M, McGrath K, Brooks AS, Lucas PW. An experimental study of hafting adhesives and the implications for compound tool technology. PLoS One. 2014;9(11):e112560 10.1371/journal.pone.0112560 25383871 PMC4226580

[23] Coles JM. Experimental Archaeology. London: Academic Press; 1979.

[24] Dibble HL, Rezek Z. Introducing a new experimental design for controlled studies of flake formation: results for exterior platform angle, platform depth, angle of blow, velocity, and force. Journal of Archaeological Science. 2009;36(9):1945–54. 10.1016/j.jas.2009.05.004

[25] Marsh EJ, Ferguson JR. Introduction In: Ferguson JR, editor. Designing Experimental Research in Archaeology. Boulder: University Press of Colorado; 2010 p. 1–12.

[26] Outram AK. Introduction to experimental archaeology. World Archaeology. 2008;40(1):1–6. 10.1080/00438240801889456

[27] ASTM. D1002-10 Standard Test Method for Apparent Shear Strength of Single-Lap-Joint Adhesively Bonded Metal Specimens by Tension Loading (Metal-to-Metal). West Conshohocken: ASTM International; 2010.

[28] ASTM. D950-03 Standard Test Method for Impact Strength of Adhesive Bonds. West Conshohocken: ASTM International; 2011.

[29] Gaillard Y, Mija A, Burr A, Darque-Ceretti E, Felder E, Sbirrazzuoli N. Green material composites from renewable resources: Polymorphic transitions and phase diagram of beeswax/rosin resin. Thermochimica Acta. 2011;521(1–2):90–7. 10.1016/j.tca.2011.04.010

[30] Charrié-Duhaut A, Porraz G, Cartwright CR, Igreja M, Connan J, Poggenpoel C, et al First molecular identification of a hafting adhesive in the Late Howiesons Poort at Diepkloof Rock Shelter (Western Cape, South Africa). Journal of Archaeological Science. 2013;40(9):3506–18. 10.1016/j.jas.2012.12.026

[31] Villa P, Pollarolo L, Degano I, Birolo L, Pasero M, Biagioni C, et al A Milk and Ochre Paint Mixture Used 49,000 Years Ago at Sibudu, South Africa. PLoS ONE. 2015;10(6):e0131273 10.1371/journal.pone.0131273 26125562 PMC4488428

[32] d’Errico F, Backwell L, Villa P, Degano I, Lucejko JJ, Bamford MK, et al Early evidence of San material culture represented by organic artifacts from Border Cave, South Africa. Proceedings of the National Academy of Sciences of the United States of America. 2012;109(33):13214–9. 10.1073/pnas.1204213109 22847420 PMC3421171

[33] Lombard M. Distribution Patterns of Organic Residues on Middle Stone Age Points from Sibudu Cave, Kwazulu-Natal, South Africa. The South African Archaeological Bulletin. 2004;59(180):37–44. 10.2307/3889241

[34] Rots V. Hafting and raw materials from animals. Guide to the identification of hafting traces on stone tools. Anthropozoologica. 2008;43(1):43–66.

[35] Iovita R, Schönekeß H, Gaudzinski-Windheuser S, Jäger F. Projectile impact fractures and launching mechanisms: results of a controlled ballistic experiment using replica Levallois points. Journal of Archaeological Science. 2014;48:73–83. 10.1016/j.jas.2013.01.031

[36] Lombard M. Direct evidence for the use of ochre in the hafting technology of Middle Stone Age tools from Sibudu Cave. Southern African Humanities. 2006;18(1):57–67.

[37] Pétillon J-M, Bignon O, Bodu P, Cattelain P, Debout G, Langlais M, et al Hard core and cutting edge: experimental manufacture and use of Magdalenian composite projectile tips. Journal of Archaeological Science. 2011;38(6):1266–83. 10.1016/j.jas.2011.01.002

[38] Moss EH, Newcomer MH. Reconstruction of Tool Use at Pincevent: Microwear and Experiments In: Cahen D, editor. Tailler! Pourquoi faire: Préhistoire et technologie lithique, Recent Progress in Microwear Studies. Studia Praehistorica Belgica. II. Tervuren: Musée Royal de L’Afrique Centrale; 1982 p. 289–312.

[39] Barton RNE, Bergman CA. Hunters at Hengistbury: Some Evidence from Experimental Archaeology. World Archaeology. 1982;14(2):237–48.

[40] Wadley L, Williamson B, Lombard M. Ochre in hafting in Middle Stone Age southern Africa: a practical role. World Archaeology. 2004;26:19–34.

[41] Rots V, Van Peer P, Vermeersch PM. Aspects of tool production, use, and hafting in Palaeolithic assemblages from Northeast Africa. Journal of Human Evolution. 2011;60(5):637–64. 10.1016/j.jhevol.2011.01.001 21392816

[42] Gibson NE, Wadley L, Williamson BS. Microscopic residues as evidence of hafting on backed tools from the 60 000 to 68 000 year-old Howiesons Poort layers of Rose Cottage Cave, South Africa. Southern African Humanities. 2004;16(1):1–11.

[43] Wadley L, Williamson B, Lombard M. Ochre in hafting in Middle Stone Age southern Africa: a practical role. Antiquity. 2004;78(301):661–75.

[44] Norlin L-H. Tall oil Ullmann's Encyclopedia of Industrial Chemistry. Weinheim: Wiley; 2005.

[45] Broughton B, Gower M. Measurement Good Practice Guide No. 47: Preparation and Testing of Adhesive Joints. Teddington: National Physical Laboratory; 2001.

[46] Brockmann W, Geiß PL, Klingen J, Schröder B, Mikhail B. Adhesive Bonding: Materials, Applications and Technology. Weinheim: Wiley; 2009.

[47] Callister WDJ, Rethwisch DG. Materials Science and Engineering: An Introduction. USA: Wiley; 2010.

[48] Shea JJ, Brown KS, Davis ZJ. Controlled experiments with Middle Paleolithic spear points: Levallois points In: Mathieu JR, editor. Experimental Archaeology: Replicating Past Objects, Behaviors, and Processes. 1035. Oxford: Archaeopress; 2002 p. 55–72.

[49] Sato C. Impact behaviour of adhesively bonded joints In: Adams RD, editor. Adhesive Bonding: Science, Technology and Applications. Cambridge: Woodhead Publishing Limited; 2005 p. 164–88.

[50] Girard M, Gaillard Y, Burr A, Darque-Ceretti E, Felder E. Nanoindentation of bio-sourced adhesive 75% rosin/25% beeswax: Experimental results and modelisation. Mechanics of Materials. 2014;69(1):185–94. 10.1016/j.mechmat.2013.10.005

[51] Clark JD. Interpretations of prehistoric technology from ancient Egyptian and other sources. Part II: Prehistoric arrow forms in Africa as shown by surviving examples of the traditional arrows of the San Bushmen. Paléorient. 1975;3:127–50.

[52] Pope ST. Yahi archery. American Archaeology and Ethnology. 1918;13(3):103–52.

[53] Rots V, Van Peer P. Early evidence of complexity in lithic economy: Core-axe production, hafting and use at Late Middle Pleistocene site 8-B-11, Sai Island (Sudan). Journal of Archaeological Science. 2006;33(3):360–71. 10.1016/j.jas.2005.08.002

[54] Pawlik AF, Thissen JP. Hafted armatures and multi-component tool design at the Micoquian site of Inden-Altdorf, Germany. Journal of Archaeological Science. 2011;38(7):1699–708. 10.1016/j.jas.2011.03.001

[55] Cunningham ML. Becoming fluent in gum arabic. Food Product Designs. 2011;21(2):1–2.

[56] Imam SH, Bilbao-Sainz C, Chiou B-S, Glenn GM, Orts WJ. Biobased adhesives, gums, emulsions, and binders: current trends and future prospects. Journal of Adhesion Science and Technology. 2012;27(18–19):1972–97. 10.1080/01694243.2012.696892

[57] Kennedy JF, Phillips GO, Williams PA. Gum arabic: Royal Society of Chemistry; 2011.

[58] Flindt C, Al-Assaf S, Phillips GO, Williams PA. Studies on acacia exudate gums. Part V. Structural features of Acacia seyal. Food Hydrocolloids. 2005;19(4):687–701. 10.1016/j.foodhyd.2004.09.006

[59] Hassan EA, Al-Assaf S, Phillips GO, Williams PA. Studies on Acacia gums: Part III molecular weight characteristics of Acacia seyal var. seyal and Acacia seyal var fistula. Food Hydrocolloids. 2005;19(4):669–77. 10.1016/j.foodhyd.2004.09.004

[60] Mhinzi GS, Mghweno LAR, Buchweishaija J. Intra-species variation of the properties of gum exudates from two Acacia species of the series Gummiferae. Food Chemistry. 2008;107(4):1407–12. 10.1016/j.foodchem.2007.09.069

