# Supplementary material for: Lap Shear and Impact Testing of Ochre and Beeswax in Experimental Middle Stone Age Compound Adhesives
Source: PLoS One. 2016 Mar 16;11(3):e0150436. doi: 10.1371/journal.pone.0150436 (PMC4794155; doi:10.1371/journal.pone.0150436)
Supplement: S1 Table — All ingredients were purchased from Kalverringdijk 29, 1509BT Zaandam, NL. Tel: +31(0)75 621 0477. Website: http://www.verfmolendekat.com/webshop/ (DOCX) [file pone.0150436.s001.docx]

| **Material** | **Supplier** |
| --- | --- |
| Pine rosin (colophonium) | Verfmolen De Kat |
| Acacia gum (Arabische gom brokjes) | Verfmolen De Kat |
| Beeswax (bijenwas korrels) | Verfmolen De Kat |
| Red ochre (Luyckse rode oker) | Verfmolen De Kat |
